# The Proximal Medial Sural Nerve Biopsy Model: A Standardised and Reproducible Baseline Clinical Model for the Translational Evaluation of Bioengineered Nerve Guides

**DOI:** 10.1155/2014/121452

**Published:** 2014-06-02

**Authors:** Ahmet Bozkurt, Sabien G. A. van Neerven, Kristl G. Claeys, Dan mon O'Dey, Angela Sudhoff, Gary A. Brook, Bernd Sellhaus, Jörg B. Schulz, Joachim Weis, Norbert Pallua

**Affiliations:** ^1^Department of Plastic Surgery, Reconstructive and Hand Surgery, Burn Centre, Medical Faculty, RWTH Aachen University Hospital, Pauwelsstraße 30, 52074 Aachen, Germany; ^2^Department of Neurology, Medical Faculty, RWTH Aachen University, 52074 Aachen, Germany; ^3^Institute of Neuropathology, RWTH Aachen University Hospital, 52074 Aachen, Germany; ^4^JARA, Translational Brain Medicine, 52074 Aachen, Germany; ^5^Clinical Trial Center Aachen (CTC-A), Medical Faculty, RWTH Aachen University Hospital, 52074 Aachen, Germany

## Abstract

Autologous nerve transplantation (ANT) is the clinical gold standard for the reconstruction of peripheral nerve defects. A large number of bioengineered nerve guides have been tested under laboratory conditions as an alternative to the ANT. The step from experimental studies to the implementation of the device in the clinical setting is often substantial and the outcome is unpredictable. This is mainly linked to the heterogeneity of clinical peripheral nerve injuries, which is very different from standardized animal studies. In search of a reproducible human model for the implantation of bioengineered nerve guides, we propose the reconstruction of sural nerve defects after routine nerve biopsy as a first or baseline study. Our concept uses the medial sural nerve of patients undergoing diagnostic nerve biopsy (≥2 cm). The biopsy-induced nerve gap was immediately reconstructed by implantation of the novel microstructured nerve guide, Neuromaix, as part of an ongoing first-in-human study. Here we present (i) a detailed list of inclusion and exclusion criteria, (ii) a detailed description of the surgical procedure, and (iii) a follow-up concept with multimodal sensory evaluation techniques. The proximal medial sural nerve biopsy model can serve as a preliminarynature of the injuries or baseline nerve lesion model. In a subsequent step, newly developed nerve guides could be tested in more unpredictable and challenging clinical peripheral nerve lesions (e.g., following trauma) which have reduced comparability due to the different nature of the injuries (e.g., site of injury and length of nerve gap).

## 1. Introduction

Injury to peripheral nerves leads to loss of motor sensory, and autonomic functions and is associated with a substantial risk of developing secondary complications. Peripheral nerve injuries not only impair patient quality of life, but also have substantial socioeconomic impact because the patients are often unable to work and require lifelong medical treatment and support [1, 2].

To date, autologous nerve transplantation (ANT) is generally accepted as the clinical gold standard for the reconstruction of overcritical peripheral nerve defects, where a tensionless direct nerve coaptation is not possible. In such cases, donor sensory nerves are usually used as autologous nerve transplants [3, 4]. However, despite innovative microsurgical techniques and extensive knowledge on peripheral nerve regeneration, functional nerve recovery is often partial and unsatisfactory [5, 6]. Even excellent coaptation techniques with exact matching of proximal and distal nerve stump fascicles do not guarantee full recovery of nerve function. Furthermore, ANT has been associated with loss of sensitivity and comorbidity at the donor site (e.g., painful neuroma formation). Another problem is the limited availability of donor nerves. Thus, there is a great demand for alternatives to ANT and much effort has been spent in developing alternative repair strategies [3].

Over recent decades, several nerve guide concepts have been developed, using natural and synthetic, resorbable or nonresorbable materials. A large number of* in vitro* and* in vivo* preclinical studies have been performed to assess the potential of bioengineered nerve conduits as alternatives to ANT using animal models [3, 7]. Subsequent substantial advances in experimental repair strategies (e.g., in material sciences including matrix design, topography and, surface functionalisation) have provided sufficient background knowledge to develop conduits for future clinical use. Such conduits require approval by the US Food and Drug Administration (FDA) or from the European Union with a Conformité Européenne (or CE) certification [8] for clinical application. Unfortunately, a direct comparison of the various nerve guide developments and with ANT is limited. The step from basic, experimental studies to the device implementation in the clinical setting is often substantial and unpredictable. This is mainly due to the heterogeneous nature of human peripheral nerve injuries, being particularly distinct from the standardised lesions used in experimental studies [9–12]. Such animal models have two major advantages: firstly, the rat sciatic or median nerve models are the most widespread animal models for peripheral nerve regeneration. The location and size of the nerve gaps in these models can be adjusted or calibrated according to the study goals. These parameters, along with the regeneration period are the most critical influencing factors that influence the degree of functional recovery. Secondly, animal models are, to a certain extent, comparable, reproducible, and reliable with established and clear cut analytical techniques (i.e., histology, functional tests, and electrophysiology) [9, 10, 13, 14]. Yet, all these advantages are absent in a clinical domain, making it challenging to reliably compare and evaluate the effectiveness of different nerve guides in the clinic. This might be the major reason for the lack of comparability of the FDA and CE approved nerve guides [8]. The range of lesion severities amongst the wide range of peripheral nerves (e.g., cranial nerves, brachial and lumbar plexus nerves, and their branches) creates enormous variability in clinical studies. Moreover, the lack of control over nerve gap size and the level of injury (e.g., proximal versus distal nerve injury) further hinder direct comparison between the different studies [8].

**Figure 1:**
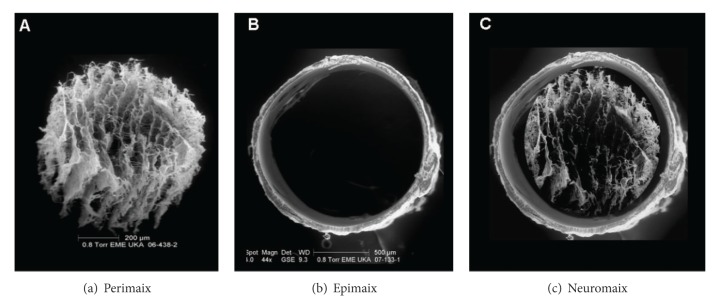
Scanning electron microscopy (SEM) of the two-component nerve guide Neuromaix (c), consisting of an outer hollow conduit (b) and inner microstructured nerve guide with longitudinal pore channels (a).

**Figure 2:**
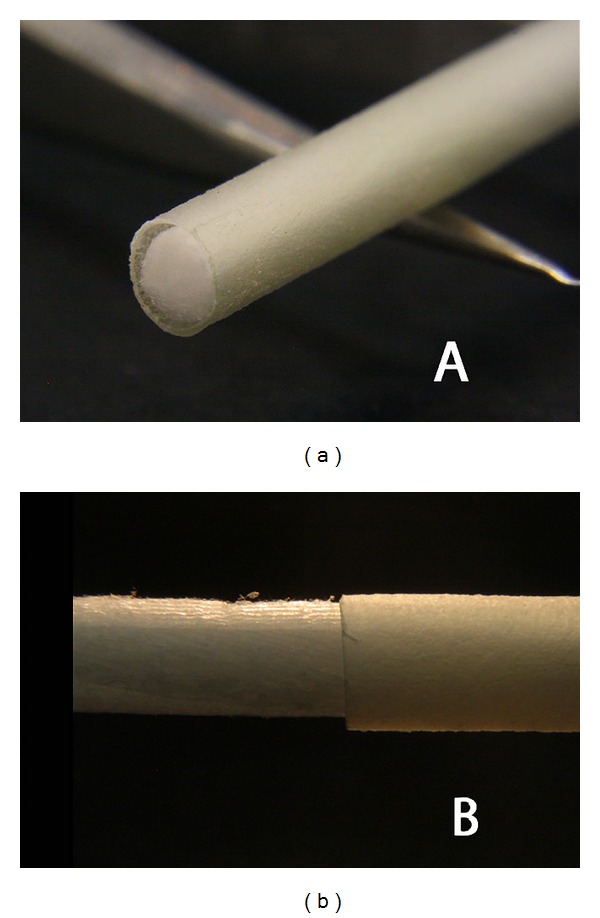
Macroscopic examples (a)-(b) of the 4 cm long Neuromaix nerve guide.

In search of a reproducible, standardised, and baseline human nerve injury model for the implantation of the recently developed peripheral nerve guide, Neuromaix (Figures 1 and 2) [15–17], we employed the proximal medial sural nerve biopsy to test issue of nerve guide safety and effectiveness. Here, we present detailed patient inclusion and exclusion criteria, the surgical procedure, and follow-up techniques for multimodal sensory outcomes. This clinical model is a safe, standardised, reproducible, and valid concept for assessing novel bioengineered peripheral nerve guides. It may not only serve as a first step in providing baseline information about the safety and performance of nerve guides, but it also clearly depends on the neuropathological condition of the patient and its influence of nerve fibre regeneration. The results from the present proximal medial sural nerve biopsy model may provide the basis for testing newly developed nerve guides under more challenging peripheral nerve injury conditions with different gaps sizes and varying location of nerve injuries.

## 2. Patients and Methods 

### 2.1. Patients

A total of 11 patients undergoing routine diagnostic nerve biopsy were implanted with the bioengineered nerve guide, Neuromaix (Figures 1 and 2) (Matricel GmbH, Herzogenrath, Germany), with the permission of the local ethical review committee and the Federal Institute for Drugs and Medical Devices (Bundesinstitut für Arzneimittel und Medizinprodukte, BfArM) (see “Perepair-”study on http://www.clinicaltrials.gov/). Patients were referred by the Neuromuscular Clinic of the Neurological Department at the RWTH Aachen University Hospital with medical assignment of unclear neuro- and/or myopathies. Patients were selected after careful neurologic history and physical examination, nerve conduction studies, and appropriate diagnostic workup for (e.g., for vasculitis, see Table 1). All patients voluntarily participated in the present study. Prior to the patients' participation in the clinical trial, a consent form was signed and personally dated by both patient and surgeon (A.B., the first author of this paper). The medical briefing included verbal and written information about possible specific complications (i.e., foreign body reactions and the prospect of success) and general complications (i.e., wound healing problems, haematoma, and necrosis).

### 2.2. Screening: Inclusion and Exclusion Criteria

We developed the following inclusion and exclusion criteria shown in Table 1.

### 2.3. Nerve Guide: Neuromaix

All patients were treated with Neuromaix (Matricel GmbH, Herzogenrath), a novel bioengineered nerve guide specifically designed for the reconstruction of peripheral nerve defects (Figures 1 and 2). Neuromaix is a collagen-based, two-component nerve guide with an outer “shell” hollow conduit (Epimaix) and an inner “core” sponge-like nerve guide (Perimaix). The latter is a microstructured three-dimensional scaffold containing numerous longitudinally orientated guidance channels with dimensions (approximately 50 *μ*m diameter) resembling natural endoneurial tubes [15]. The microchannels of the Perimaix collagen scaffold were created by a patented unidirectional freezing process developed by Matricel GmbH (Herzogenrath, Germany) [15–17]. For the present study, Neuromaix nerve guides were provided with a standard length of 4 cm and a diameter of 3 mm and were shortened according to the required length of the respective nerve defect (Figures 1 and 2).

## 3. The Proximal Medial Sural Nerve Biopsy Model Surgery: Operative Technique

**Figure 3:**
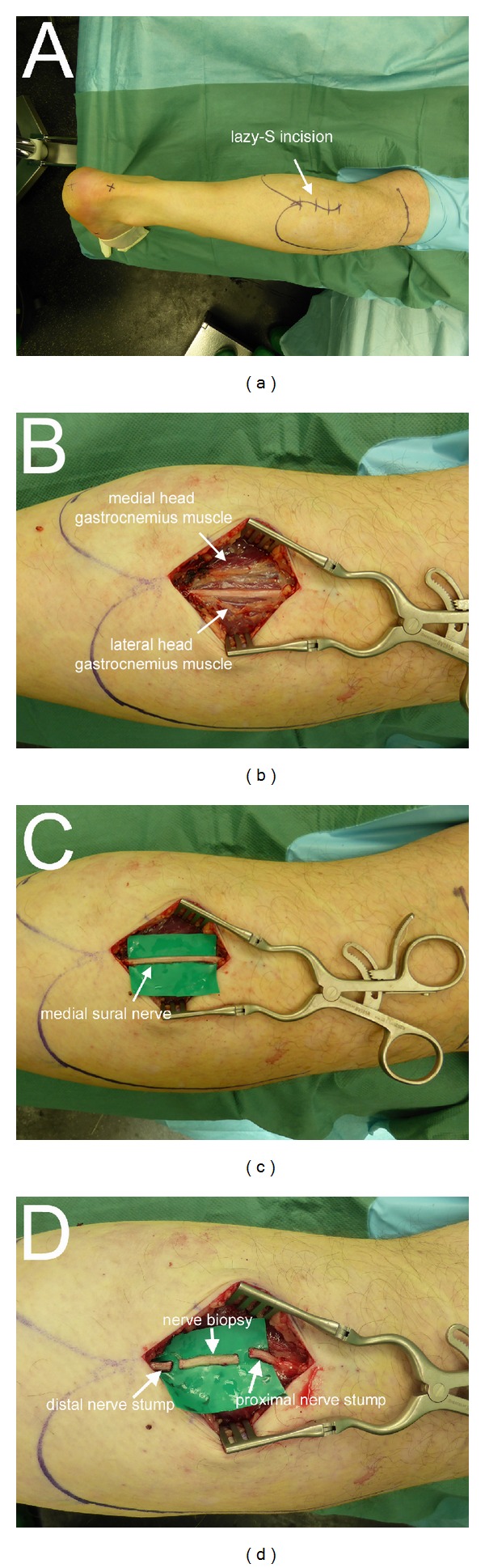
Operative technique (Part I). Marking the outline of the medial and lateral heads of the gastrocnemius muscle, the popliteal crease, the insertion of the Achilles tendon, and the lazy-S-line of incision on the proximal part of the lower leg (a). Exposure of the medial sural nerve between the medial and lateral heads (white arrows) of the gastrocnemius muscle before (b) and after (c) external neurolysis. Excision of a 2 cm nerve biopsy segment for neuropathological examination (d).

**Figure 4:**
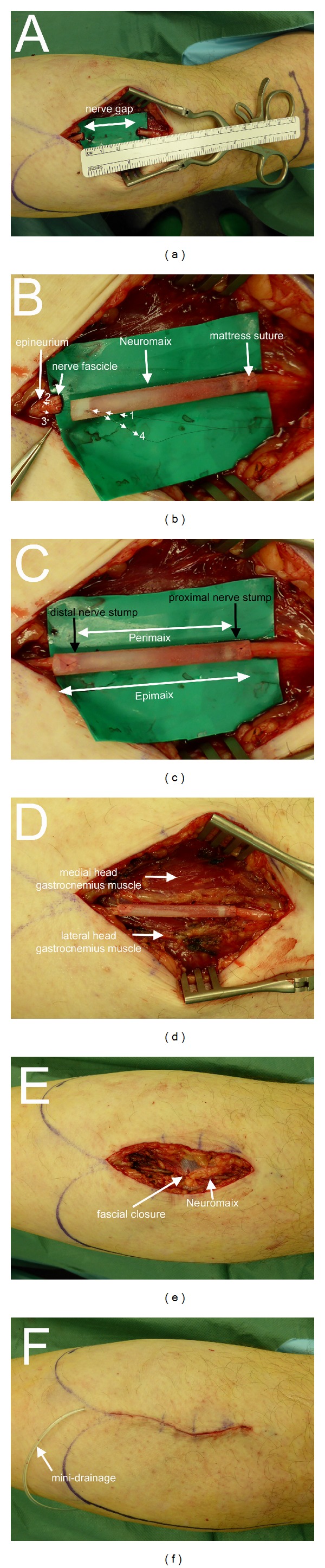
Operative technique (Part II). Gap between the proximal and distal nerve stumps resulting from excision of a 2 cm nerve biopsy (a). Please note that the resulting nerve gap (approximately 3 cm) is larger than the length of the nerve biopsy (approximately 2 cm) due to the elastic retraction of the nerve stumps. Implantation of the nerve guide using the entubulization technique by means of 8.0 mattress sutures. Note the start of the mattress suture at the side of the nerve guide, positioning the knot on the nerve guide with the following sequence of needle penetration: 1 Neuromaix → 2 epineurium → 3 epineurium →4 Neuromaix (b)-(c). After completion of the entubulization procedure, the implanted nerve guide is located in its wound bed between the medial and lateral heads of the gastrocnemius muscle (d). Wound closure in anatomic layers (e) after insertion of a minivacuum drainage system (f).

**Figure 5:**
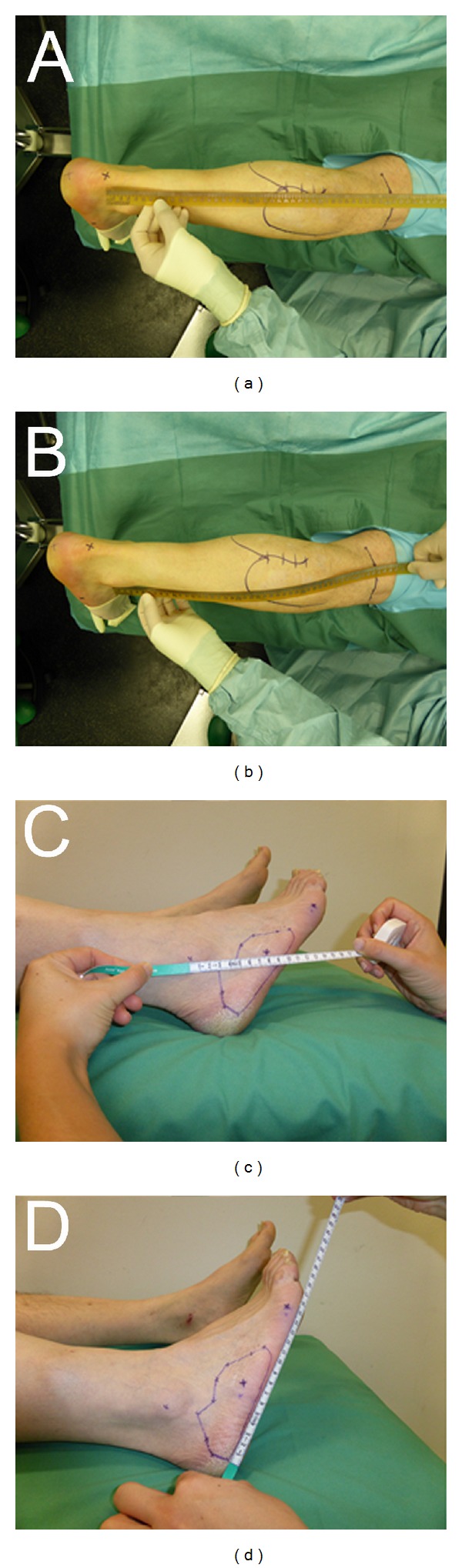
Landmarks for the documentation of sensory reinnervation. Relationship between the site of nerve guide implantation (possible regeneration along the course of the medial sural nerve) and the popliteal crease, insertion of the Achilles tendon (a) and lateral malleolus (b). Note the circumscribed hypesthetic area at the lateral aspect of the foot (c)-(d). This area is also assessed in relation to the lateral malleolus and midlateral point of the fifth metatarsal bone.

The nerve biopsy was performed on an outpatient basis with the patient lying in prone position without any sedation. All patients received prophylactic intravenous antibiotic (Ampicillin/Sulbactam, 3 g). The palpable raphe between the lateral and medial gastrocnemius was identified by asking the patient to perform plantar- and dorsiflexion against resistance. All patients were treated under local anaesthesia with a subcutaneous injection of approximately 10 mL of Xylonest 1% supplemented with noradrenaline (1 : 200.000). A 4-5 cm lazy-S incision was performed along the midline axis of the posterior lower leg between the lateral and medial gastrocnemius muscle at the musculotendinous transition (Figure 3(a)). A tourniquet was not necessary. After incision of the skin and fascia and identification of the lesser saphenous vein, the medial sural nerve and sural artery were identified in the subfascial plane between the medial and lateral head of the gastrocnemius muscle (Figure 3(b)). Using magnifying glasses, an atraumatic external neurolysis was performed (Figure 3(c)). The nerve was then flushed with approximately 2 mL of the local anaesthetic in order to abolish the pain associated with the excision of the sural nerve. A 2 cm segment of the medial sural nerve was excised without crushing the nerve stumps and transferred to the Institute of Neuropathology in normal saline solution (Figure 3(d)). The bleeding from the proximal and distal stumps was treated by covering the nerve ends with a saline soaked compress. The nerve guide was prepared by immersion in sterile normal saline solution. When the bleeding from the nerve stumps had stopped, the nerve guide was implanted by means of the entubulization technique [18]. Neuromaix was interposed between the nerve stumps (Figure 4(a)) with an overlap of 2-3 mm at each end. At each end, a single 8-0 horizontal interrupted mattress suture (Ethilon 8.0, Ethicon Inc, Somerville, USA) was used to secure the outer epineurium of the nerve stumps within Neuromaix nerve guide. We preferred to start the mattress suture at the side of the nerve guide, positioning the knot on the nerve guide with the following sequence of the needle penetration: Neuromaix → Epineurium → Epineurium →Neuromaix (Figure 4(b)). The nerve stumps could then be gently drawn into the open ends of the nerve guide. For further protection against dislocation, the mattress suture was flanked by two accompanying single 8.0 stitches, taking care that only the outer epineurium was grasped (Figures 4(b)–4(d)). If a muscle biopsy was required it was harvested from the gastrocnemius muscle. Before wound closure, the proximal and distal ends of the medial sural nerve were noted in relation to defined landmarks, that is, the popliteal crease, the lateral malleolus, the insertion of the Achilles tendon, and the midlateral point of the fifth metatarsus for later documentation and sensory evaluation (see Figure 5 and Table 3). The closure of healthy soft tissue over the nerve guide was critical to achieve proper wound healing (Figures 4(e) and 4(f)). After insertion of a minivacuum drainage system, the incision was closed in anatomical layers using inverted single Monocryl 2.0 and 3.0 stitches and a continuous intracutaneous skin suture using Prolene 3.0 (Ethilon, Ethicon Inc, Somerville, USA) (Figures 4(c) and 4(d)). Finally a sterile dressing was applied. Postoperative management consisted of removal of the drainage system at day 1 or day 2, and removal of sutures at day 14. Antithrombosis prophylaxis was performed by subcutaneous injection of low molecular weight heparin (Clexane, 20 mg) for patients with risk factors (e.g., obesity, use of contraceptives, or cigarette smoking) and the use of forearm crutches was recommended for the first 10–12 days.

### 3.1. Multimodal Sensory Testing

Sensory testing of the lower leg and the lateral aspect of the foot will be performed in a multimodal approach (Table 2) pre- and postoperatively: directly after surgery, and at 1, 3, 6, and 12 months. Testing will be performed on the ipsilateral and contralateral (untreated) sides for intraindividual comparison (Figure 5) and estimation of the nerve regeneration in relationship to landmarks (Table 3). For sensory evaluations, the following landmarks and measurements will be documented: popliteal crease, lateral malleolus, insertion of the Achilles tendon, and midlateral point of the 5th metatarsus (Figure 5).

## 4. Preliminary Clinical Results

**Figure 6:**
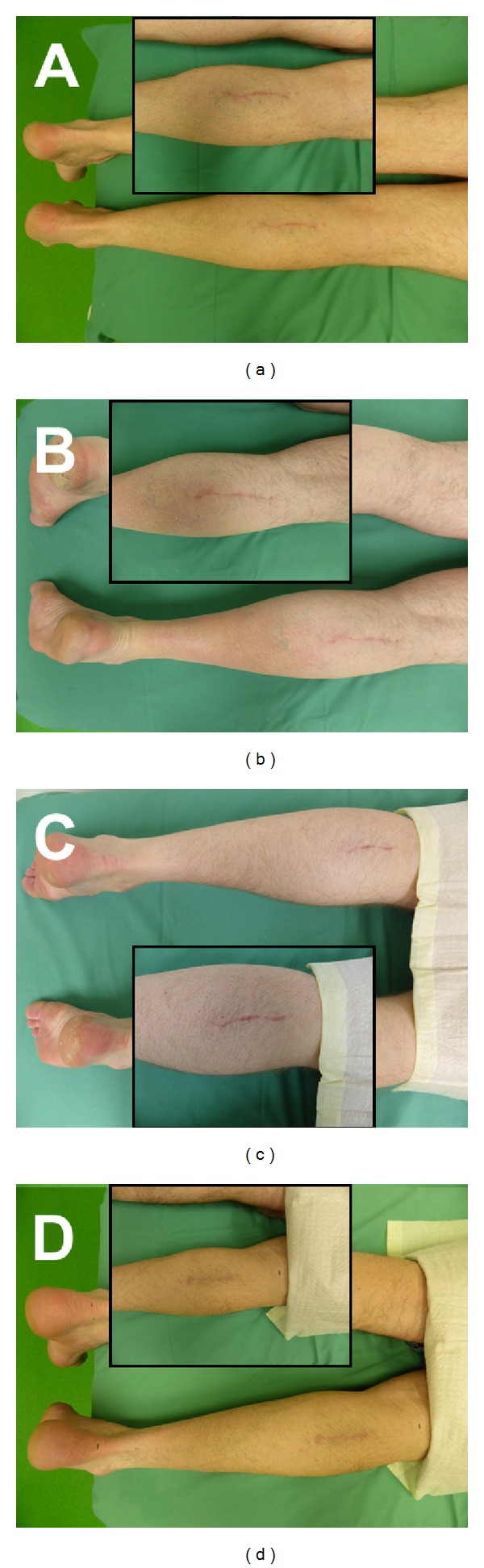
Representative images of wound healing at the site of the nerve biopsy in the lower leg of 4 patients (a)–(d), 3 months after surgery. There were no signs of delayed wound healing or other complications (no foreign body or allergic reaction, no infection, etc.) at the 3 month endpoint of the safety phase.

Between July 2013 and January 2014, a total of *n* = 11 patients (male = 9, female: 2) with a mean age of 58.36 years (range = 49–67 years) were enrolled in the present study with diagnostic sural nerve biopsy (biopsy length: 2 cm) and subsequent Neuromaix implantation. Seven patients had an additional muscle biopsy (gastrocnemius muscle: *n* = 6, lateral vastus muscle: *n* = 1). All patients recovered well without any wound healing problems (see Figure 6). There was no need for any kind of revision. Sutures could be removed after 14–21 days.

**Figure 7:**
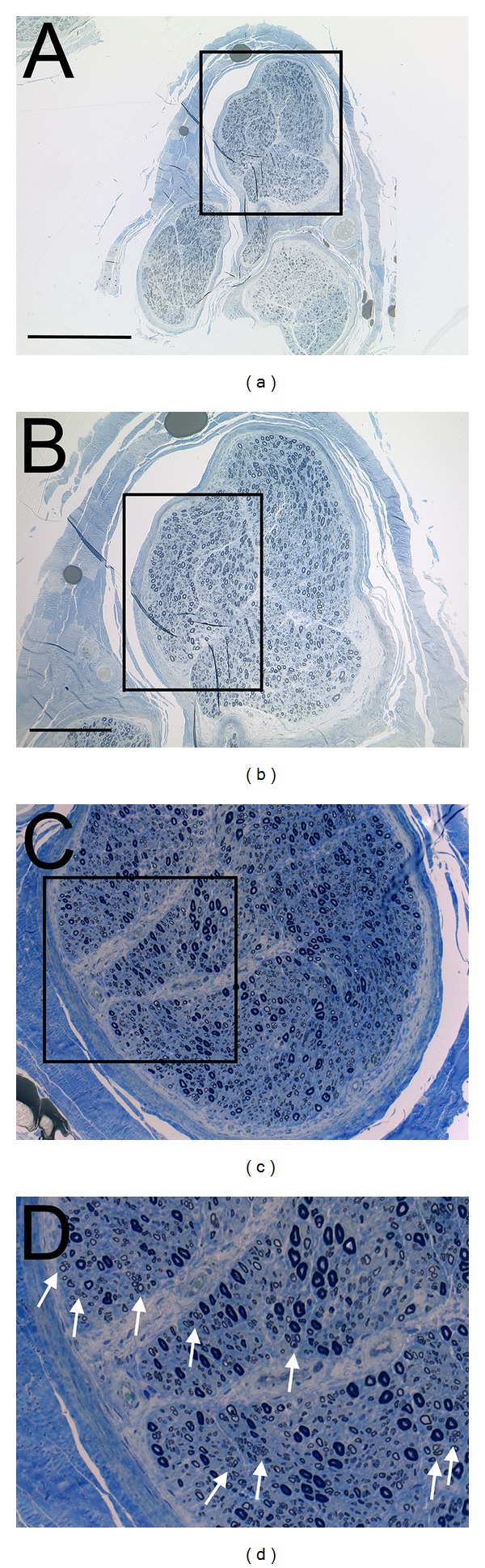
Representative histology (semithin section, toluidine blue staining) of a patient with an inflammatory neuritis (see Table 4) (a)–(d). Note parallel appearance of large diameter, myelinated axons (approximately 50% reduced in number when compared to a normal sural nerve), and the large number of regenerating fibers arranged in clusters (see white arrows in (d)).

Histopathological examination revealed an inflammatory neuritis in 6 patients (i.e., 54,5%) and an idiopathic or hereditary polyneuropathy in five patients (i.e. 45,5%) (Table 4). Most importantly, histopathology revealed clusters of regenerating axons in all 11 patients (i.e., 100%) (Figure 7).

## 5. Discussion

The ANT is still the clinical gold standard for the reconstruction of overcritical sized peripheral nerve defects [4]. However, depending on the initial injury pattern, functional outcome is often only partial or unsatisfactory [6, 19]. The ANT is associated with comorbidities at the donor site and the availability of donor nerves is limited. Therefore, a wide range of nerve conduits have been developed from diverse natural and synthetic polymers (e.g., collagen, chitosan, or polycaprolactone) in combination with sophisticated surface modifications [20]. These conduits have been evaluated in preclinical, experimental studies for their efficacy in supporting nerve regeneration. According to Chalfoun and colleagues [21], the ideal bioengineered nerve guide should meet several critical requirements: it should protect the regenerating axons from the surrounding environment and provide a permissive environment to Schwann cells (which can be transplanted or can migrate into the nerve guide from the proximal and distal nerve stumps). Furthermore, it is assumed that nerve regeneration is promoted by the increased accumulation of neurotrophic factors within the lumen of the conduit [22, 23]. Apart from autologous veins [24–26], clinical alternatives to the ANT are either processed (decellularized) nerve allografts [27] or hollow nerve conduits with FDA and/or CE approval [8]. The use of nerve conduits is much faster and simpler in clinical practice (“off the shelf approach” with unlimited material available) than harvesting a donor nerve that demands extra surgery with subsequent increased operation time and risk of complications [28]. However, such clinically approved alternatives to ANT are still not widely accepted and are viewed critically [8]. There is clearly the need for a safe, standardised, and reproducible human nerve lesion model for assessing and comparing the range of currently available and newly developed peripheral nerve guides. As described in the present study, the proximal medial sural nerve biopsy model offers a number of advantages. The patients enrolled for a diagnostic nerve biopsy would normally be left with an untreated nerve defect. However, implantation of a nerve guide into the medial sural nerve defect could facilitate the elimination or reduction of (i) possible painful neuroma formation and/or (ii) loss of sensitivity at the lateral aspect of the foot, which are the most common long-term problems after complete or partial sural nerve biopsy [29–31]. Three previous publications have followed the same line of logic but with some differences. Flores and colleagues reconstructed the biopsied sural nerve with autologous saphenous vein grafts [24]. They assumed that the time for recovery was not shortened but the quality of regeneration mediated reinnervation was considered superior than in patients without nerve repair. More recently, and independent of our ongoing study, Radtke and colleagues correctly hypothesised that biopsy-mediated sural nerve defects might be used as a regeneration model for testing the efficacy of nerve conduits in humans [32]. Schoeller and colleagues [31] performed nerve biopsies at the lateral malleolus by resecting a 10 mm segment and directly suturing both nerve ends after neurolysis. We do not recommend performing the nerve biopsy with subsequent reconstruction with conduits at the lateral malleolus for three striking reasons: firstly, although the “traditional approach” at the lateral malleolus is easier to perform and has a shorter distance to the target area (lateral aspect of the foot), the incidence of comorbidities (i.e., wound dehiscence, wound infection, healing problems, loss of sensation, chronic pain, and formation of painful neuromas) is significantly higher at the lateral malleolus when compared to the proximal lower leg “modified approach” that provides improved soft tissue coverage [33]. Secondly, a muscle biopsy is often performed simultaneously to the nerve biopsy, but the posterolateral aspect of the leg is not an optimal location for such a procedure, as the muscles located there are predominantly tendonous. Thirdly, the traditional approach at the lateral malleolus involves the complete sural nerve “common sural nerve,” while the modified approach at the proximal lower leg involves a part of the sural nerve “medial sural nerve,” partially preserving sensation in the target territory of the sural nerve [33, 34]. Thus, although the traditional approach is easier and has a shorter distance for the axon regeneration, the present modified approach is substantially safer with respect to wound healing (as it has a better blood supply) and exhibits a lower incidence of wound infection or dehiscence, preventing dislocation of the reconstructed nerve. Furthermore, in our experience and in that reported by others nerve biopsies should ideally be longer than 10 mm in order to enable multiple processing techniques (including paraffin wax embedding, cryostat sections, and semithin sections of resin embedded material for electron microscopy) [16, 18]. Moreover, the detection of focal lesions in many neuropathies, such as in vasculitis- or amyloidosis-associated neuropathies, often requires sampling over extended lengths of nerve [35, 36].

Some aspects of the present concept require specific consideration. The patients included in this study presented with particular neurological symptoms that required a nerve biopsy (see Table 1 for inclusion and exclusion criteria). These patients can clearly not be regarded as comparable with “healthy” or “normal” patients who have suffered from a traumatic peripheral nerve injury. However, the diagnosis of neuropathy does not exclude peripheral nerve regeneration. On the contrary, axonal clusters with short internodes have regularly been found in nerve biopsies, indicating that some degree of axonal degeneration and regeneration is possible (see also Figure 7). Furthermore, in some neuropathies regenerating axon clusters are so numerous that fiber diameter histograms reveal an absolute increase in the number small diameter, myelinated fibres. This must be differentiated from neuropathies that are characterized by degenerating neuronal cell bodies and the lack of regenerating axonal clusters [37].

The current model should only be considered as a first step in the translational approach for the implantable bioengineered nerve guides. The presented model is safe, standardized, reproducible, and valid but limited concept in testing and evaluation of peripheral nerve guides. It may serve as a first step providing baseline information regarding the safety and performance of a nerve guide under relatively comparable conditions, which is depending on the regenerative capacity of the respective patients with a neuropathy. The results from the presented proximal medial sural nerve model in nerve biopsy patients may serve as a preliminary study for testing the respective nerve guide under more challenging conditions which are mostly hampered by the two factors: size of the nerve gap and site of the injury.

## 6. Conclusion

The aim of the present (ongoing) study is to present a safe, effective, standardised, and reproducible human model for nerve guide implantation as a first step in enabling the transition of newly developed nerve guides into the clinical setting (from bench to bedside). The modified proximal medial sural nerve biopsy approach provides an improvement in standardisation of lesion parameters, thus presenting itself as a valid technique in the assessment of peripheral nerve guides in humans.

## Figures and Tables

**Table 1:** Patient selection: inclusion and exclusion criteria.

Inclusion criteria	Exclusion criteria
(i) Patients between 18 and ≤70 years of age (ii) Patients with clinical and electrophysiological diagnosis of peripheral neuropathy with the indication for a nerve biopsy to establish the cause of the neuropathy	(i) Alcohol-related polyneuropathy (PNP) (ii) Paraneoplastic PNP (iii) Ongoing immunosuppressive therapy (iv) Malignant tumour (v) Peripheral vascular disease (vi) Collagenous diseases (vii) Diabetes mellitus (viii) Chronic venous insufficiency (ix) Deep vein thrombosis (x) Skin diseases of the lower extremity (xi) Coagulopathy or anticoagulant therapy (xii) Pregnancy (xiii) Infectious diseases (i.e., HIV, hepatitis)

**Table 2:** List of sensory testing.

Multimodal sensory testing		
Sensory modality	Testing strategy	Unit/scale
Clinical evaluation	(Delayed wound healing, redness, swelling, pus, seroma, hypertrophic scarring, allergy, and foreign body sensation)	
(1) Nociception	Visual Analog Scale (VAS) used for pain estimation	Value: 0–10
(2) Loss of sensation (hypesthesia)	Measurement and photo documentation of the area on the lateral aspect of the foot with loss of sensation	Value: cm^2^
	Sharp-blunt discrimination	Value: yes/no
(3) Tinel's sign	Palpation revealing Tinel's sign and documentation of location in relation to the landmarks	Value: relationship to landmarks in cm
(4) Spatial resolution	Static and moving/dynamic two-point discrimination	Value: s/m 2-PD in mm
(5) Pressure	Semmes-Weinstein monofilament test	Value: monofilament strength in g
(6) Thermoception	Cold-warm discrimination	Value: yes/no
(7) Vibration	128 Hz tuning fork	Value: scale from 0–8

**Table 3:** Landmarks and distances.

**Landmarks and distances**		
from	to	distance (in cm)
**Intraoperative**		
PC	IOAT	
PC	LM	
PC	MOMV	
PNS	PC	
PNS	IOAT	
PNS	LM	
PNS	MOMV	
DNS	PC	
DNS	IOAT	
DNS	LM	
DNS	MOMV	
MOS	PC	
MOS	IOAT	
MOS	LM	
MOS	MOMV	
LONB		
LOS		
NPLIDE		
AWLOSLF		

**Postoperative follow-up**		
AWLOSLF		
LOTS	PC	
LOTS	IOAT	
LOTS	LM	
LOTS	MOMV	

**Abbreviation**		

IOAT	Insertion of Achilles tendon	
PC	Popliteal crease	
LM	Lateral malleolus	
MOMV	Midpoint of metatarsus V	
PNS	Proximal nerve stump	
DNS	Distal nerve stump	
MOS	Midpoint of scar	
LONB	Length of nerve biopsy	
NPLIDE	Nerve gap length in dorsal extension	
LOS	Length of scar	
AWLOSLF	Area with loss of sensibility lateral foot	
LOTS	Location of Tinel's sign	

**Table 4:** Patient data.

Number	Gender	Age	Site	Additional muscle biopsy	Histopathologic diagnosis	Histopathology: regenerating cluster
1	M	56 y	Right	—	Neuritis (inflammatory)	Yes
2	F	61 y	Right	—	Idiopathic PNP	Yes
3	F	65 y	Right	Gastrocnemius muscle	Idiopathic PNP	Yes
4	M	49 y	Right	Gastrocnemius muscle	Neuritis (inflammatory)	Yes
5	M	52 y	Right	—	Hereditary PNP	Yes
6	M	61 y	Left	Gastrocnemius muscle	Neuritis (inflammatory)	Yes
7	M	59 y	Left	Lateral vastus muscle	Hereditary PNP	Yes
8	M	54 y	Left	Gastrocnemius muscle	Neuritis (inflammatory)	Yes
9	M	61 y	Left	Gastrocnemius muscle	Hereditary PNP	Yes
10	M	57 y	Right	Gastrocnemius muscle	Neuritis (inflammatory)	Yes
11	M	67 y	Left	—	Neuritis (inflammatory)	Yes
